# ISG15 facilitates cellular antiviral response to dengue and west nile virus infection *in vitro*

**DOI:** 10.1186/1743-422X-8-468

**Published:** 2011-10-13

**Authors:** Jianfeng Dai, Wen Pan, Penghua Wang

**Affiliations:** 1 Institute of Biology and Medical Sciences, Jiangsu Key Laboratory of Infection and Immunity, Soochow University, Suzhou 215123 P.R. China; 2 Section of Infectious Diseases, Department of Internal Medicine, Yale University School of Medicine, New Haven, Connecticut, USA

**Keywords:** ISG15, Dengue Virus, West Nile Virus, ISGylation

## Abstract

**Background:**

Dengue virus (DENV) and West Nile virus (WNV), close siblings of the *Flaviviridae *family, are the causative agents of Dengue hemorraghic shock or West Nile meningoencephalitis respectively. Vaccines against these two flaviviruses are currently unavailable. Interferon- Stimulated Gene 15 (*ISG15*), encoding an ubiquitin-like protein, is significantly induced by type I interferons or viral infections. Its roles in viral infections, however, vary with viruses, being either anti- or pro-viral. The exact roles of ISG15 in DENV and WNV infections remain unknown. In the current study, we evaluated the relevancies of ISG15 to DENV and WNV infection of a mouse macrophage cell line RAW264.7.

**Findings:**

Quantitative PCR showed that mouse *Isg15 *was dramatically induced in DENV or WNV- infected RAW264.7 cells compared with non-infected cells. *Isg15 *and two other Jak-Stat related genes, *Socs1 *and *Socs3*, were silenced using siRNA mediated RNA interference. The intracellular DENV and WNV loads, as determined by quantitative PCR, were significantly higher in *Isg15 *silenced cells than control cells. The expression levels of interferon beta 1 (*Ifnb1*) were increased significantly in *Isg15*, *Socs1 *or *Socs3 *siRNA treated cells. Further investigation indicated that protein modification by ISG15, so called ISGylation, was significantly enhanced in DENV-infected cells compared to that in non-infected cells.

**Conclusions:**

These findings suggest that ISG15 plays an anti-DENV/WNV function via protein ISGylation.

## Findings

Dengue virus (DENV) and West Nile virus (WNV) are two major flaviviruses that can infect human via mosquito bites [1]. Both viruses are (+) sense single strand RNA viruses and replicate in the cytoplasm of their host cells. DENV infection causes Dengue fever (DF) or Dengue hemorrhagic fever (DHF), and the latter may lead to death of the patient [2]. WNV is a re-emerging zoonotic pathogen of medical importance. In humans, WNV infection may cause life-threatening meningoencephalitis or long-term neurologic sequelae [3]. Vaccines against both DENV and WNV are currently not available, thus much efforts are needed to elucidate the mammalian host anti-viral mechanisms against these two flaviviruses in vitro and in vivo [1-3].

ISG15 is one of the first identified Interferon-Stimulated- Genes (ISGs). The expression of ISG15 can be induced by type I interferons (IFNs), viral infections and LPS, suggesting that ISG15 is a broad spectrum of stress response gene [4,5]. The protein product of ISG15 shows a significant sequence homology to ubiqutin, and this protein can conjugate to numerous cellular proteins via isopeptide bonds. This kind of protein modification is called ISGylation, which also utilizes a three step reaction similar to protein ubiquitination. The specific enzymes for ISGylation are UBE1L (E1), UBCH8 (E2) and HERC5 (E3), respectively. Like ubquitination, ISGylation is reversible, mediated by deISGylation enzymes, such as UBP43. Notably, expression of UBE1L, UBCH8, HERC5, and UBP43 is also induced by IFN [4-7]. Till now, more than 200 cellular proteins were identified as the substrates of ISG15, while the functional consequences of reversible ISG15 modification of most target proteins are still largely unknown [8,9].

A strong induction of ISG15 in response to IFN treatment or viral infections implies an antiviral role for ISG15, yet surprisingly initial studies using vesicular stomatitis virus (VSV) and lymphocytic choriomeningitis virus (LCMV) demonstrated that ISG15 or UBE1L are dispensable for anti-viral immune responses in mice [10,11]. Nevertheless, more emerging work has shown that ISG15 plays an antiviral role against many viral pathogens *in vitro *and *in vivo*, including influenza A/B, herpes simplex virus type 1, murine gamma herpes virus, HIV and etc. [12-18]. ISG15 achieves its antiviral role by conjugating to target proteins including both host proteins and viral proteins and altering their functions. For example, ISG15 can conjugate to host anti-viral protein IRF3 and thus stabilize IRF3 by inhibiting its interaction with PIN1, a protein that promotes IRF3 ubiquitination and degradation [19]. ISG15 was also found to modify the influenza A non-structural protein NS1A, and inhibit its immunosuppressive function [15]. Little work has been done on the physiological relevancies of ISG15 to flavivirus infections [14,20]. Surprisingly though, a recent study even showed that ISG15 is favorable of hepatitis C virus infection [21]. It is though not clear why ISG15 "likes" some viruses, but "dislikes" some others, it is imperative to understand ISG15 function case-by-case. We hereby investigated the physiological role of ISG15 in DENV and WNV infection in a mouse macrophage cell line.

### *Isg15 *is upregulated upon DENV or WNV infection

**Figure 1:**
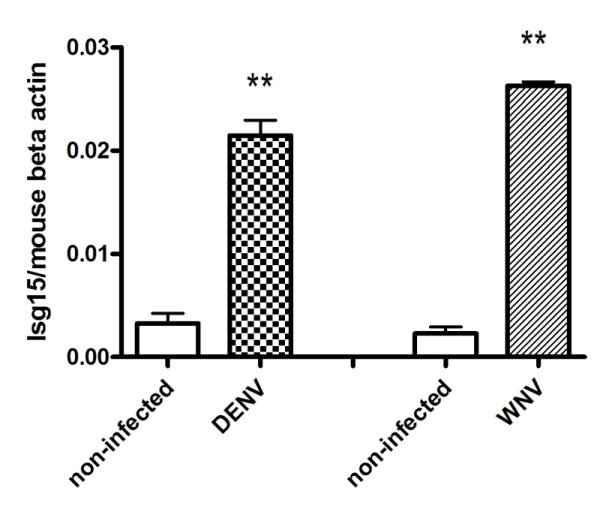
***Isg15 *is upregulated upon DENV (A) or WNV (B) infection**. The virus burdens were analyzed by measuring the virus E gene copy using quantitative RT-PCR, and normalized to mouse beta actin gene. Results are expressed as the mean + the SEM. * *p *< 0.05 and ** *p *< 0.01. Representative results from at least 3 independent experiments.

Mouse macrophage-like cell line RAW264.7 can be efficiently infected by DENV and WNV, and is an established *in vitro *model for the study of host innate immune response to these viruses. By using a quantitative PCR based small cDNA array (SABiosciences, Frederick, MD), we measured the expression of Jak-Stat signaling pathway related genes in DENV (DENV 2 New Guinea C strain) or WNV (NY99 strain) infected RAW264.7 cells. A number of genes were found up- or down-regulated upon virus infection (Additional File 1). Among them, *Isg15 *was upregulated 9-fold in DENV infected cells, and 69-fold in WNV infected cells compared with non-infected controls (Additional File 1). An independent quantitative PCR (Figure 1) further confirmed the upregulation (6.5-fold and 11.5-fold, respectively upon DENV or WNV infection) of *Isg15 *indentified from PCR array.

### *Isg15 *activities repress DENV and WNV infection in RAW264.7 cells

**Figure 2:**
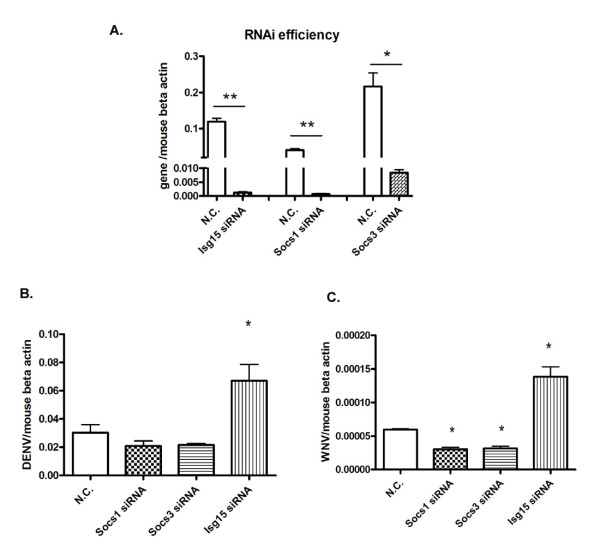
**The viral burden is increased in *Isg15 *silenced cells**. A) RNAi efficiency of specific genes. The mRNA levels of a specific gene were analyzed by Q-RT-PCR and normalized to mouse beta-actin gene. B and C) DENV (B) or WNV (C) burdens in RAW264.7 cells after RNAi silencing. Results are expressed as the mean + the SEM. * *p *< 0.05 and ** *p *< 0.01. Representative results from at least 3 independent experiments.

To study the specific role of *Isg15 *in DENV and WNV infection, a siRNA based RNA interference study was performed in RAW264.7 cells. siRNAs specific for gene *Isg15, Socs1, Socs3 *from Jak-Stat pathway (Table 1) or scrambled control (N.C.) were delivered into RAW264.7 cells respectively via electroporation (*Socs1 *and *Socs3*, negative regulators of Jak-Stat signaling were included as controls in this study). 24 hrs after siRNA transfection, cells were challenged by DENV (MOI = 1.0) or WNV (MOI = 1.0) for another 24 hrs, respectively. Cells were then harvested and total RNA and cDNA were made according to standard protocols. The genes were silenced efficiently (Figure 2A) as analyzed by quantitative PCR using gene specific primers (Table 1); the expression of *Isg15*, *Socs1 *and *Socs3 *were decreased 97, 59 and 24-fold, respectively, upon specific siRNA treatment (Figure 2A). The intracellular viral loads, in terms of the transcript levels of the envelop gene (E), were quantified by quantitative PCR and normalized to mouse beta actin gene. As shown in Figure 2B and 2C, the DENV load was increased 2.1-fold (*p *< 0.05) and WNV load increased 2.4-fold (*p *< 0.05) in *Isg15 *silenced cells, respectively, compared with control cells. These data suggest that *Isg15 *has an anti-viral activity against both DENV and WNV in murine macrophage-like cells. Furthermore, WNV burden was significantly decreased in *Socs1 *(2.0-fold) *or Socs3 *(1.8-fold) silenced cells compared to controls (Figure 2C) and a trend of reduction in DENV burden was also found in *Socs1/3 *silenced cells (Figure 2B). These results suggest that the Jak-Stat pathway controls DENV and WNV infection in RAW264.7 cells. Silencing the negative regulators of Jak-Stat pathway, *Socs1 *or *Socs3*, resulted in an increased Jak-Stat activity and decreased viral replication. On the other hand, viral replication was increased when *Isg15 *was silenced. This finding confirmed an anti-DENV/WNV role for *Isg15 *in an *in vitro *model.

**Table 1:** siRNA and oligo-primer sequences for this study:

**No**.	Sequence (5'-3')	Note
1	CAGUGAUGCUAGUGGUACAtt	siRNA seq for mouse gene *Isg15*

2	GCAUCCCUCUUAACCCGGtt	siRNA seq for mouse gene *Socs1*

3	GCAUCUUUGUCGGAAGACUtt	siRNA seq for mouse gene *Socs3*

4	AGAGGGAAATCGTGCGTGAC	Forward primer for Quantitative-PCR of mouse beta-actin

5	CAATAGTGATGACCTGGCCGT	Reverse primer for Quantitative-PCR of mouse beta-actin

6	CATTCCAAGTGAGAATCTCTTTGTCA	Forward primer for Quantitative-PCR of DENV E gene

7	CAGATCTCTGATGAATAACCAACG	Reverse primer for Quantitative-PCR of DENV E gene

8	TTCTCGAAGGCGACAGCTG	Forward primer for Quantitative-PCR of WNV E gene

9	CCGCCTCCATATTCATCATC	Reverse primer for Quantitative-PCR of WNV E gene

10	GGAACGAAAGGGGCCACAGCA	Forward primer for Quantitative-PCR of mouse Isg15 gene

11	CCTCCATGGGCCTTCCCTCGA	Reverse primer for Quantitative-PCR of mouse Isg15 gene

12	GCCGCAGCATTAAGTGGGGGC	Forward primer for Quantitative-PCR of mouse Socs1 gene

13	GGTCTCCAGCCAGAAGTGGGAGG	Reverse primer for Quantitative-PCR of mouse Socs1 gene

14	TGAGCCATCTTGGAGCCCAGGT	Forward primer for Quantitative-PCR of mouse Socs3 gene

15	TTGGCTGTGTTTGGCTCCTTGTGT	Reverse primer for Quantitative-PCR of mouse Socs3 gene

### *Ifnb1 *expression is upregulated when *Isg15*, *Socs1 *or *Socs3 *is silenced

**Figure 3:**
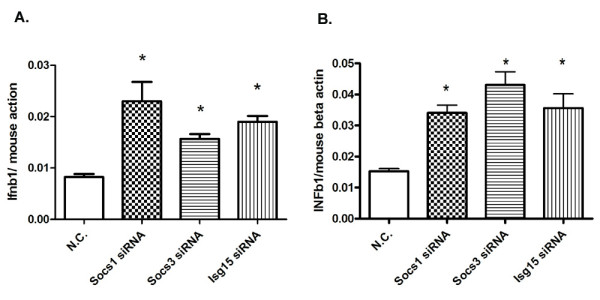
***Ifnb1 *expression in virus infected cells after gene silencing**. *Ifnb1 *gene expression level was measured by quantitative RT-PCR and normalized to mouse beta-actin gene. Results are expressed as the mean + the SEM. * *p *< 0.05 and ** *p *< 0.01. Representative results from at least 3 independent experiments.

We further measured the expression of representative type I interferon (*Ifnb1*) in these cells. In both DENV and WNV infected cells, the *Ifnb1 *transcript levels were higher (up to 2~3 fold) in *Socs1 *or *Socs3 *silenced groups (Figure 3), which is consistent with their inhibitory function on the Jak-Stat and other anti-viral pathways. Surprisingly, *Ifnb1 *expression was also significantly increased in *Isg15 *silenced cells infected with DENV (2.9-fold) and WNV (2.3-fold). It is not clear how ISG15 impacts type I IFN expression in this study though; one explanation is ISG15 could be an inhibitor of type I IFN production. Alternatively, an increase in *ifnb1 *expression is a result of secondary effect of increased viral burden (Figure 3).

### Protein ISGylation is enhanced in DENV infected cells

**Figure 4:**
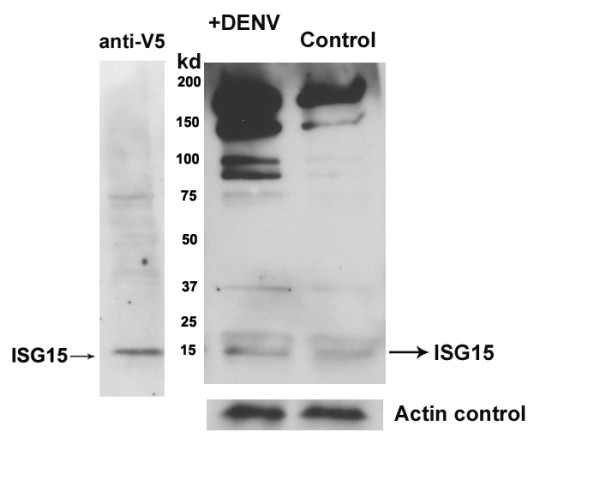
**ISGylated protein profiles from DENV infected or non-infected RAW264.7 cells**. A) Western blots of V5-tagged ISG15 in RAW 264.7 cells. B) Western blots of ISG15-conjugated proteins from DENV infected or non-infected cells.

To further understand the antiviral mechanism of *Isg15*, we analyzed protein ISGylation profiles of DENV infected or non-infected RAW264.7 cells. The cDNA for mouse *Isg15 *were cloned into pcDNA3.1-V5-His vector and transfected into RAW264.7 cells. To create the stable cell line, the cells were selected using G418 for 2 weeks, and verified by Western blotting using an anti-V5 tagged antibody (Figure 4A). These cells were then infected with DENV for 24 hrs with a MOI of 1.0. ISG15-conjugated proteins were then purified from DENV infected or non-infected cells by affinity chromatography using an anti-V5 monoclonal antibody crosslinked to protein A/G resin. Western blots show that after DENV infection, the concentrations of ISG15-conjugated proteins were dramatically increased compared to non-infected controls (Figure 4B), suggesting ISG15 inhibits viral replication by modifying viral or cellular proteins. Similar results were observed with human monocyte cell lines (K562) and mouse embryo fibroblast cells (MEFs) (data not shown).

Protein modification by ubiquitin and ubiquitin-like proteins, including ISG15, SUMO and Nedd8, Fat 10, has been shown to be important for many cellular processes, such as cell cycle, stress response and immune response [4]. ISG15 has been shown to be upregulated upon type I interferon stimulation, microbial infections and tumor growth [4,5,22,23]. Expression of ISG15 is reported to be controlled by transcriptional factors Stat, NFkb, p53 and JNK [4]. These studies suggest that ISG15 is generally a stress-responsive gene.

The role of ISG15 and its conjugation activity in viral infections has been intensively studied recently, though controversial results have been reported with different viruses [4,7]. One study shows that *Isg15 *is largely dispensable for the type I IFN-induced antiviral response against VSV and LCMV in mice [10]. While some other work indicate that *Isg15*^-/- ^or *Ube1L*^-/- ^(ISG15 E1) mice are more susceptible to many viral infections, such as influenza A/B, herpes simplex virus and SNV, than WT mice [4]. ISG15 can modify a number of important antiviral proteins, including RIG-1, IRF3, MDA-5, Mx1, PKR, STAT1 and JAK1, and potentially influence their functions [4,19,24]. However, Functional consequences of this reversible modification are still mysterious. For examples, ISGylation of RIG-I negatively regulates RIG-I signaling and leads to a reduction of IFN production [24]; while ISGylation of IRF3 prevents the ubiquination and degradation of IRF3 during DNV infection [19]. ISG15 can inhibit the ubiquitination of Gag and Tsg101 of HIV-1, disrupts their interaction, and prevents the virus assembles [17], while ISG15 also promotes HCV infection in an *in vitro *model [21]. Generally speaking, ISGylation does not lead to degradation of target proteins, in stead, ISGylation stabilizes the target proteins by inhibiting their ubiquitination and degradation [4].

On the other hand, viruses may also block the antiviral activity of ISG15 [4]. For example, nario-viruses and arterviruses encode ovarian-tumor (OTU)-domain containing proteases, which can deconjugate ISG15. Overexpression of OUT proteases antagonizes the antiviral effect of ISG15 [25].

Since ISG15 plays distinct role in different viral infection model, we use DENV and WNV to explore the function of ISG15 in these two vector-borne RNA viruses. Our results suggest that ISG15 has an antiviral activity against these two flaviviruses. And the antiviral activity of ISG15 likely relies on its protein-modifying activity. Our results also confirmed the inhibitory effect of *Socs1 *and *Socs3*, on antiviral mechanisms such as the Jak-Stat pathway. Future directions would be characterizing the target proteins of ISGylation during DENV or WNV infection, and indentifying novel anti-flavivirus molecules and/or new mechanisms mediated by ISGylation. These studies may significantly increase our knowledge on the pathogenesis of flavivirus infection and advance the development of novel therapeutics or drugs against these life-threatening viruses.

## Abbreviations

ISG15: Interferon Stimulate Gene 15; DENV: Dengue virus; WNV: West Nile virus; *Ifnb1*: Interferon beta 1; *Socs*: Suppressor of cytokine signaling

## Competing interests

The authors declare that they have no competing interests.

## Authors' contributions

JD and PW designed the experiments and prepared the manuscript. JD and WP performed all the experiments. All authors read and approved the final manuscript.

## Supplementary Material

Additional file 1 **The expression of Jak-Stat pathway related gene during DENV or WNV infection**. The expression of genes from Jak-Stat signaling pathway were measured using a quantitative PCR based small cDNA array (SABiosciences, Frederick, MD). A number of genes were found up- or down-regulated upon DENV or WNV infection. Click here for file
