# Supplementary material for: ISG15 facilitates cellular antiviral response to dengue and west nile virus infection in vitro
Source: Virol J. 2011 Oct 13;8:468. doi: 10.1186/1743-422X-8-468 (PMC3215395; doi:10.1186/1743-422X-8-468)
Supplement: Additional file 1 — The expression of Jak-Stat pathway related gene during DENV or WNV infection. The expression of genes from Jak-Stat signaling pathway were measured using a quantitative PCR based small cDNA array (SABiosciences, Frederick, MD). A number of genes were found up- or down-regulated upon DENV or WNV infection. [file 1743-422X-8-468-S1.DOC]

**Additional File 1: The expression of Jak-Stat pathway related gene during DENV or WNV infection.**

|  | fold change Vs. non-infected | |  | fold change Vs. non-infected | |
| --- | --- | --- | --- | --- | --- |
| GENE | DENV | WNV | GENE | DENV | WNV |
| Sh2b2 | -3.12 | 1.15 | Irf1 | -1.82 | 4.59 |
| Bcl2l1 | -3.16 | 1.41 | Irf9 | 1.44 | 3.03 |
| Cebpb | -2.13 | 1.52 | Jak1 | 1.05 | -1.23 |
| Crk | -2.99 | 1.07 | Jak2 | 2.41 | 2.46 |
| Csf1r | -1.49 | 2.30 | Mmp3 | 3.68 | -2.99 |
| Cxcl9 | -1.89 | 5.66 | Mpl | 6.32 | 21.11 |
| F2 | -3.27 | -2.64 | Nos2 | -1.58 | 1.59 |
| F2r | 6.87 | -2.46 | Nr3c1 | -1.14 | -1.62 |
| Fas | 1.62 | 14.93 | Oas1a | 1.07 | 6.96 |
| Fcgr1 | -1.59 | 5.28 | Prl | -1.00 | 6.96 |
| Isg15 | 9.05 | 68.59 | Prlr | -1.00 | 22.63 |
| Gata3 | -2.10 | 3.25 | Saa3 | -3.34 | 9.85 |
| Gbp1 | 3.97 | 25.99 | Sh2b1 | -2.83 | -1.74 |
| Il10ra | -3.01 | 1.62 | Sit1 | -1.53 | -3.48 |
| Il10rb | -2.91 | -1.32 | Smad1 | 4.32 | -1.00 |
| Il2rg | -2.07 | 3.48 | Smad2 | 3.56 | 1.23 |
| Il4 | -3.27 | -8.00 | Socs1 | 1.57 | 5.66 |
| Il4ra | -1.25 | 1.32 | Socs2 | -1.09 | 1.87 |
| Il6st | -2.06 | -1.52 | Socs3 | -2.14 | 8.57 |

* Mean value of fold changes from two independent experiments were shown. The shadow indicates the genes we focused on this study.
